# Elevated lymphotoxin-α (TNFβ) is associated with intervertebral disc degeneration

**DOI:** 10.1186/s12891-020-03934-7

**Published:** 2021-01-13

**Authors:** Zhu Guo, Chensheng Qiu, Christina Mecca, Yang Zhang, Jiang Bian, Yan Wang, Xiaolin Wu, Tianrui Wang, Weiliang Su, Xianglin Li, Wei Zhang, Bohua Chen, Hongfei Xiang

**Affiliations:** 1 Department of Orthopedic Surgery, the Affiliated Hospital of Qingdao University, Qingdao, 266000 Shandong China; 2 Department of Orthopedic Surgery, Qingdao Municipal Hospital (Group), Qingdao, 266011 Shandong China; 3 Department of Anesthesiology, Medical College of Wisconsin, Milwaukee, WI 53226 USA; 4 Department of Spine Surgery, Weifang People’s Hospital, Weifang, 261041 Shandong China; 5 Qingdao Eye Hospital of Shandong First Medical University, Qingdao, 266000 Shandong China; 6 School of Medical Imaging, Bin Zhou Medical University, Yantai, 264003 China

**Keywords:** Intervertebral disc degeneration, Lymphotoxin-α, TNFβ, Inflammatory response, Cytokine

## Abstract

**Background:**

Intervertebral disc degeneration (IVDD) is a primary cause of degenerative disc diseases; however, the mechanisms underlying the degeneration remain unclear. The immunoinflammatory response plays an important role in IVDD progression. The inflammatory cytokine lymphotoxin-α (LTα), formerly known as TNFβ, is associated with various pathological conditions, while its role in the pathogenesis of IVDD remains elusive.

**Methods:**

Real-time quantitative polymerase chain reaction (RT-qPCR), Western blotting (WB), and enzyme-linked immunosorbent assays were used to assess the levels of LTα in human nucleus pulposus (NP) tissues between degeneration and control groups. The plasma concentrations of LTα and C-reactive protein (CRP) were compared between healthy and IVDD patients. Rat primary NP cells were cultured and identified via immunofluorescence. Methyl-thiazolyl-tetrazolium assays and flow cytometry were used to evaluate the effects of LTα on rat NP cell viability. After NP cells were treated with LTα, degeneration-related molecules (Caspase-3, Caspase-1, matrix metalloproteinase (MMP) -3, aggrecan and type II collagen) were measured via RT-qPCR and WB.

**Results:**

The levels of both the mRNA and protein of LTα in human degenerated NP tissue significantly increased. Plasma LTα and CRP did not differ between healthy controls and IVDD patients. Rat primary NP cells were cultured, and the purity of primary NP cells was > 90%. Cell experiments showed inversely proportional relationships among the LTα dose, treatment time, and cell viability. The optimal conditions (dose and time) for LTα treatment to induce rat NP cell degeneration were 5 μg/ml and 48 ~ 72 h. The apoptosis rate and the levels of Caspase-3, Caspase-1, and MMP-3 significantly increased after LTα treatment, while the levels of type II collagen and aggrecan were decreased, and the protein expression levels were consistent with their mRNA expression levels.

**Conclusions:**

This study demonstrated that elevated LTα is closely associated with IVDD and that LTα may induce NP cell apoptosis and reduce important extracellular matrix (ECM) proteins, which cause adverse effects on IVDD progress. Moreover, the optimal conditions for LTα treatment to induce NP cell degeneration were determined.

**Supplementary Information:**

The online version contains supplementary material available at 10.1186/s12891-020-03934-7.

## Background

Intervertebral disc degeneration (IVDD) is well-recognized as the pathological basis of degenerative disc diseases [1]. More than 70% of the population will suffer from degenerative disc diseases in their lifetime, which places a serious burden on family and society [2].

IVDD initiates in the nucleus pulposus (NP) tissue [3]. Abnormal NP cell apoptosis and excessive extracellular matrix (ECM) degradation are considered the main causes of IVDD. The pathogenesis of IVDD is associated with many factors, including aging, heredity, immunoinflammatory responses, metabolic disease, smoking, and poor nutrition; however, the pathogenetic role of these risk factors in IVDD is still not fully understood [4–9]. Among these factors, inflammatory cytokines play a crucial role in IVDD via mediating the inflammatory response, resulting in ECM degradation and disc cell death and eventually leading to severe spinal degenerative disease [10].

The tumor necrosis factor (TNF) superfamily has 19 members [11], and the most extensively studied inflammatory cytokine associated with IVDD is TNFα [12]. Lymphotoxin-α (LTα) is another vital member of the TNF superfamily and has a crucial role in immunoinflammatory response, host defense, and immune system development [13]. Since LTα and TNFα present many similarities in terms of gene structure, protein molecular structure, and biological functions, LTα was formerly known as TNFβ. However, further studies revealed many differences between TNFβ and TNFα, especially in cell origin, secretion dynamics, signal transduction pathway, and gene expression regulation [14], which resulted in the renaming of TNFβ to LTα [14]. Recent studies suggest that LTα is closely related to immunoinflammatory-related diseases, such as rheumatoid arthritis (RA) [15] and graft-versus-host disease (GVHD) [16]. However, the relationship between LTα and IVDD has rarely been evaluated. A recent study of intervertebral disc proteomics analysis by our research team showed that LTα was closely related to age-related IVDD [17].

To further probe the role of LTα in IVDD pathogenesis, the levels of LTα in human NP tissue and plasma were determined and compared between normal controls and IVDD patients. Rat NP cells were treated with exogenous LTα, and the cell viability and degeneration-related molecules were measured to evaluate the effects of LTα on NP cells. The relationship between LTα and IVDD as well as the associated clinical significance were discussed from multiple perspectives.

## Methods

### Collection and grading of human NP tissues

Lumbar disc NP tissue samples were obtained during spinal fusion surgery from patients with idiopathic scoliosis or spinal trauma, and these patients had an average age of 27.8 ± 5.2 years old (*n* = 15, including 8 males and 7 females) and were considered the control group. The patients had no previous history of low back pain. The degree of degeneration was classified as grade I-II (Pfirrmann grading system) [18], which is usually considered clinically normal for discs [19, 20]. Lumbar disc NP tissue samples were also collected during spinal fusion surgery from patients diagnosed with lumbar spinal stenosis or discogenic low back pain, and these patients had an average age of 60.1 ± 5.7 years old (*n* = 15, including 7 males and 8 females) and were considered the degeneration group. The degree of degeneration of these patients was classified as grade IV. Magnetic resonance imaging (MRI) confirmed that the discs were degenerated and showed mild bulging without exhibiting obvious extrusion, sequestration or compression of the nerve root. All patients were free from diabetes, liver or kidney disease, tumors, immune system diseases or infections. The annulus fibrosus (AF) and cartilage endplate were separated and removed under a microscope. After resection and washing with normal saline, NP tissues were stored in liquid nitrogen. Tissue collections were carried out under sterile conditions. The clinical diagnosis was made by two spine surgeons and a radiologist.

### Blood collection and plasma C-reactive protein (CRP) detection

Venous blood specimens were obtained from healthy volunteers, who had an average age of 24.3 ± 2.1 years old (*n* = 15, including 8 males and 7 females) and were considered the control group, and the aforementioned IVDD patients (n = 15) were used as the degeneration group. MRI confirmed that the discs of the volunteers were normal. Blood samples of IVDD patients were collected before surgery. After centrifugation (10 min, 3000×*g*, 4 °C), plasma was collected and separated into two tubes. One tube was used to measure the CRP concentration at the clinical laboratory and the other sample was stored in liquid nitrogen until analysis.

### Enzyme-linked immunosorbent assay (ELISA)

The NP tissues were cut into 1 mm^3^ pieces and extracted with lysis buffer (Cloud-Clone, China). After ultrasound treatment and centrifugation, the supernatant was obtained for the assay. The levels of cytokine LTα in human NP tissues and plasma samples were determined by a LTα-specific ELISA kit (Cloud-Clone). The detection range was from 15.6 pg/ml to1000 pg/ml, and the sensitivity limit was 8 pg/ml.

### Cell culture

Rat primary NP cells were purchased from Procell Life Science & Technology (Cat No.: CP-R145, Lot No.: 36I18010601; Procell, China). The NP cells were passaged twice or three times before the experiments. In brief, four-week-old Sprague-Dawley rats from the Hubei Provincial Center for Disease Control and Prevention (Wuhan, China) were sacrificed via broken neck. The entire spine was obtained, and NP tissues were isolated from the lumbar region. The NP tissues were then cut into 1 mm^3^ pieces and digested by 0.2% Dispase II (Cat No.: D4693-1G; Sigma, USA) and 0.2% type II collagenase (Cat No.: C6885-25MG; Sigma) at 37 °C for 4 h. After a pipette was used to lightly blowing on the digested tissues 20 times, the tissues were filtered through a 200-mesh strainer. The filtrate was then collected and centrifuged (300×g, 5 min), and the seed cells were cultured in rat NP cell medium (Cat No.: CM-R145; Procell) at 37 °C under 5% CO_2_.

### Immunocytofluorescence

Immunofluorescence identification of primary rat NP cells was carried out by Procell. In brief, primary NP cells were fixed with 4% paraformaldehyde (Sinopharm, China) before permeabilization with 0.5% Triton X-100 (Beyotime, China). The cells were incubated with anti-type II collagen antibody (1:100, Cat No.: BA0533; Boster, China) at 4 °C overnight and then with Cy3-labeled secondary antibody (1:100, Cat No.: BA1032; Boster) at room temperature for 1 h. The nuclei were counterstained with 4,6-diamino-2-phenyl indole (DAPI) (Beyotime). A fluorescence microscope (BX53, Olympus, Japan) was used for image capture. The positive cells under five random high-power fields were counted under a light microscope, and the purity of NP cells was assessed by determining the type II collagen-positive cell rate.

### Methyl-thiazolyl-tetrazolium (MTT) cell viability assay

Time- and dose-dependent experiments were performed to evaluate the effects of LTα on rat NP cells. The survival rate was determined via MTT assay to identify the optimal treatment conditions. Briefly, after seeding in 96-well plates (7 × 10^3^ cells per well), P3 NP cells in the experimental and control groups were cultured to the logarithmic growth phase before treatment. To evaluate the dose dependence, different concentrations of LTα (0, 0.01, 0.1, 1, 2, 3, and 5 μg/ml) (Cat No.: 10270-HNAE; Sino Biological, China) were added to the experimental group and incubated for 72 h. To evaluate the time dependence, LTα (5 μg/ml) was added to the experimental group and incubated for 24 h, 48 h, and 72 h. Equal volumes of sterile phosphate buffered saline (PBS) were added to the control group. MTT (Sigma) solution and dimethyl sulfoxide (DMSO) (Sigma) were added in turns, and the optical density (OD) values were tested as reported in previous method [21].

### Rat NP cell treatment

To evaluate the effects of LTα on NP cells, P3 NP cells were seeded in 6-well plates (2.5 × 10^5^ cells per well) and divided into an experimental group and a control group. The NP cells were cultured to the logarithmic growth phase before treatment. LTα (5 μg/ml) and an equal volume of PBS were added to the experimental and control groups, respectively. The cells were cultured at 37 °C under 5% CO_2_ for 72 h and then collected for flow cytometry (FCM) and Western blotting (WB) analysis.

### FCM analysis of cell apoptosis

After the LTα treatment, FCM was used to evaluate cell apoptosis with an Annexin V-FITC/PI apoptosis detection kit (Vazyme, China). Briefly, after each group was treated, the cells were centrifuged, resuspended in binding buffer, and then stained with Annexin V-FITC and PI. Cells in the early and late apoptotic stages were counted.

### RNA extraction and real-time quantitative polymerase chain reaction (RT-qPCR)

Total RNA was extracted from human NP tissues with TRIzol reagent (Thermo Fisher, USA), and extracted from rat NP cells with a Takara MiniBEST Universal RNA Extraction Kit (Takara, Japan) according to the manufacturer’s guidelines. First-strand cDNA was synthesized using Hifair® II 1st Strand cDNA Synthesis SuperMix for qPCR (gDNA Digester Plus) (Yeasen Biotech, China). RT-qPCR was performed using a Bio-Rad real-time PCR system with Hieff® qPCR SYBR Green Master Mix (No Rox) (Yeasen Biotech) following the standard procedure. The sequences of primers used are shown in Table 1. The GAPDH housekeeping gene was used as the internal control.

**Table 1:** The sequences of primers of RT-qPCR

Gene Name	Forward/ Reverse	5′-3′Sequence	Size
LTα	Forward	CCTGGCTGCACTCGATGT	127 bp
	Reverse	GCGAAGGCTCCAAAGAAG	
Caspase-3	Forward	CTGGACTGCGGTATTGAG	102 bp
	Reverse	GGGTGCGGTAGAGTAAGC	
Caspase-1	Forward	CAGGAGGGAATATGTGGG	120 bp
	Reverse	AACCTTGGGCTTGTCTTT	
MMP-3	Forward	ACCTATTCCTGGTTGCTG	105 bp
	Reverse	GGTCTGTGGAGGACTTGTA	
Aggrecan	Forward	TGAAACCACCTCTGCATTCCA	96 bp
	Reverse	GACGCCTCGCCTTCTTGAA	
Type II collagen	Forward	GTCACAGAAGACCTCACGCCTC	81 bp
	Reverse	TCCACACCGAATTCCTGCTC	
GAPDH	Forward	TCAAGAAGGTGGTGAAGCAGG	115 bp
	Reverse	TCAAAGGTGGAGGAGTGGGT	

### Protein extraction and WB

Total proteins were extracted from human NP tissues and rat NP cells using RIPA lysis buffer (Beyotime), and their concentrations were determined using the BCA method (Beyotime). Subsequently, 25 μg of protein was loaded onto 8% or 12% separation gel and 5% stacking gel and subjected to SDS-PAGE. After transferring the proteins to PVDF membranes (Beyotime), the membranes were blocked in 5% nonfat dry milk with TBST and then treated with specific antibodies. The following primary antibodies were used: LTα (1:1000, Cat No.: DF6453; Affinity Biosciences, USA), type II collagen (1:1000, Cat No.: 28459–1-AP; Proteintech, China), aggrecan (1:1000, Cat No.: 13880–1-AP; Proteintech), Caspase-1 (1:1000, Cat No.: 22915–1-AP; Proteintech), Caspase-3 (1:1000, Cat No.: 19677–1-AP; Proteintech), matrix metalloproteinase (MMP)-3 (1:1000, Cat No.: 66338–1-lg; Proteintech), and GAPDH (1:5000, Cat No.: ATPA00013Rb; AtaGenix, China). HRP- conjugated goat to rabbit IgG (1:5000, Cat No.: SA00001–2; Proteintech) and HRP- conjugated goat to mouse IgG (1:5000, Cat No.: SA00001–1; Proteintech) were used as secondary antibodies. The GAPDH housekeeping protein was used as the internal control. Proteins were detected through enhanced chemiluminescence and visualized using a gel imaging system (Bio-Rad, USA). Semiquantitative analysis of the bands was performed by Image J (Image J 1.51j8, NIH, USA).

### Statistical analysis

All the experiments were performed at least 3 times (biological and technical replications). The data were expressed as the means ± standard deviations. Independent Student’s *t-*tests were used to compare two groups. A one-way analysis of variance and Tukey’s multiple comparison test were used to compare multiple groups. A *P* value of < 0.05 was considered statistically significant. GraphPad Prism 7.04 (GraphPad Software, Inc., USA) was used to perform statistical analyses.

## Results

### Degenerated human discs presented a higher Pfirrmann grade level and LTα was increased in human degenerated NP tissues

**Fig. 1:**
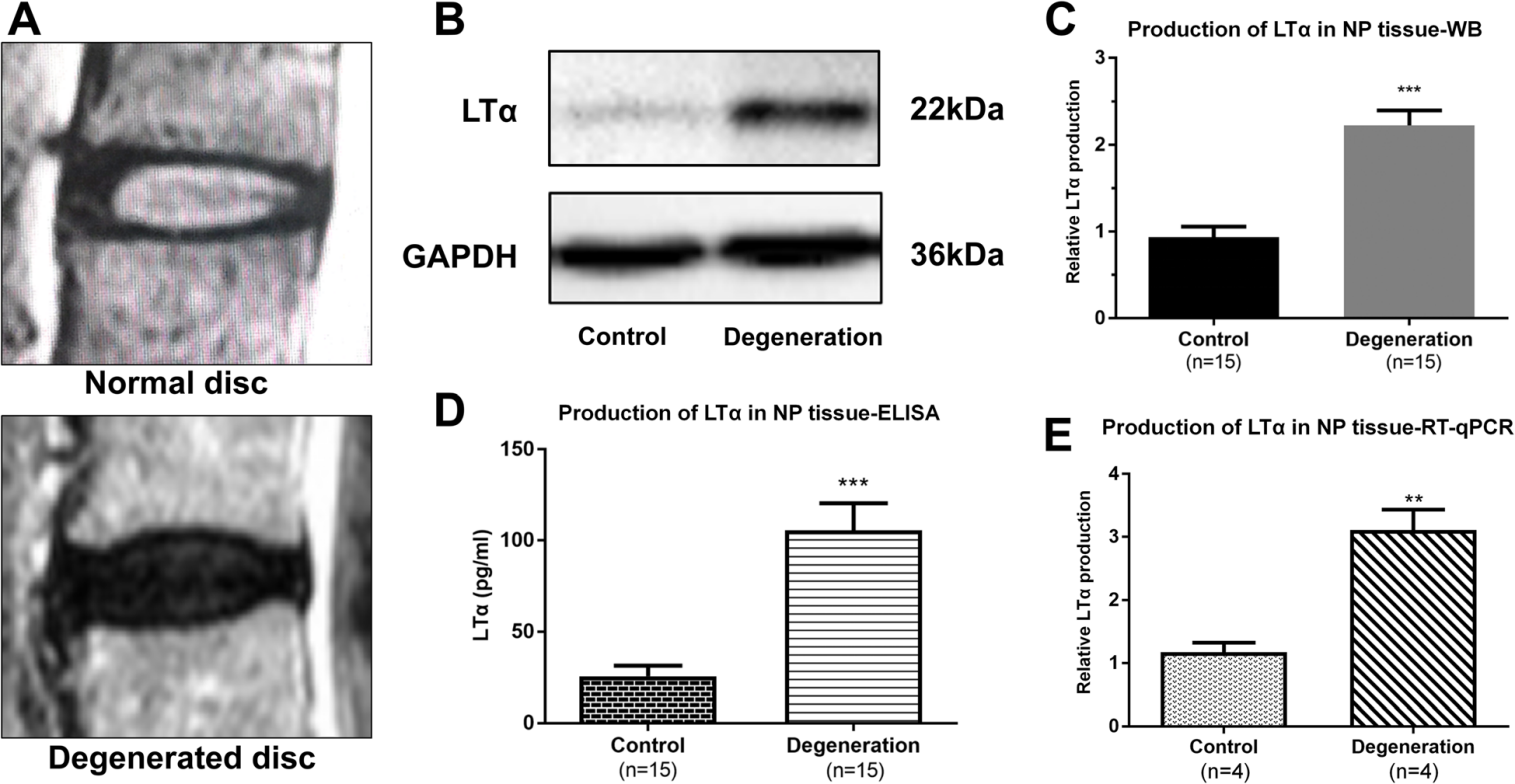
Images of intervertebral disc degeneration and detection of LTα in human NP tissues. (A) Upper panel: normal disc in a MRI T2 image; bottom panel: degenerated disc in a MRI T2 image. (B) Protein lysates from NP from the control and degeneration groups were blotted with anti-LTα and anti-GAPDH antibodies. (C) Quantitative analysis of LTα protein levels as determined in “B”. (D) Concentration of LTα in NP tissue determined by ELISA. (E) LTα mRNA production in NP tissue determined by RT-qPCR. Control group: relatively healthy disc samples from patients with idiopathic scoliosis or spinal trauma; degeneration group: degenerated disc samples from patients with lumbar spinal stenosis or discogenic low back pain. The GAPDH housekeeping gene/protein was used as the internal control. **. *p* < 0.01; ***. *p* < 0.001 (Unpaired, two-tailed Student’s *t*-test)

The T2-weighted MRI of intervertebral discs in the control group showed hyperintense signals, a clear distinction between the AF and NP, and a normal disc height. The discs were classified as grade I-II, which represented normal discs (Fig. 1a, upper panel). However, discs in the patients with degeneration showed hypointense signals, the disc heights were normal to moderately decreased, and the distinction between the AF and NP was lost. The discs were classified as grade IV, which represented degenerated (Fig. 1a, bottom panel). WB, ELISA and RT-qPCR were applied to detect the levels of LTα in human NP tissues. Significantly increased production of LTα was found in the degeneration group (Fig. 1b, c, d and e).

### Plasma LTα and CRP showed no differences between the healthy controls and IVDD patients

**Fig. 2:**
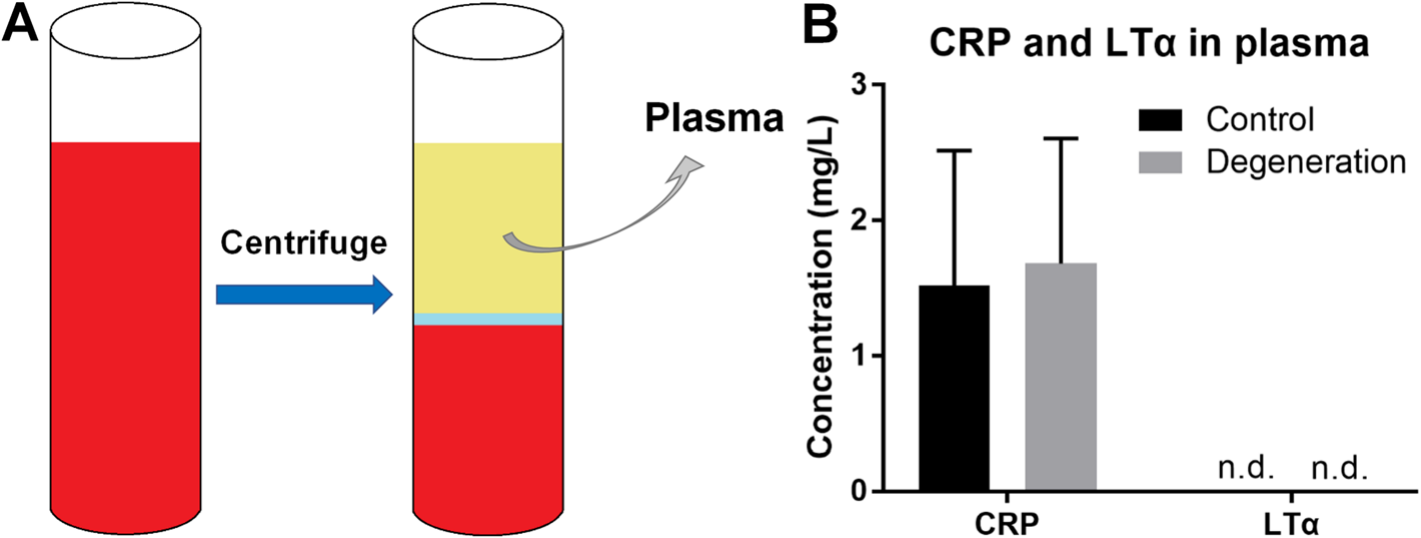
Determination of CRP and LTα in human plasma. (A) Schematic diagram of the plasma extraction. (B) The concentrations of plasma CRP in both the control and degeneration groups were in the normal range, and no significant difference was found. The concentrations of plasma LTα were determined by ELISA, and the values were low and could not be detected in both groups (n.d.: not detectable). Control group: blood specimens from healthy volunteers (*n* = 15); degeneration group: blood specimens from IVDD patients (n = 15). *p* > 0.05 (Unpaired, two-tailed Student’s *t*-test)

The inflammatory marker CRP and cytokines can be increased in the peripheral blood of patients with lumbar disc herniation and thus have important significance for clinical diagnosis and disease surveillance [22, 23]. To identify a potential biomarker of non-herniated IVDD in peripheral blood, we examined the plasma LTα and CRP levels. The concentrations of plasma CRP in both the control and degeneration groups were in the normal range, and no significant difference was found (Fig. 2b). The concentrations of LTα in both the control and degeneration groups were very low and could not be detected (Fig. 2b).

### Inflammatory cytokine LTα caused a decrease in rat NP cell viability and induced cell apoptosis

**Fig. 3:**
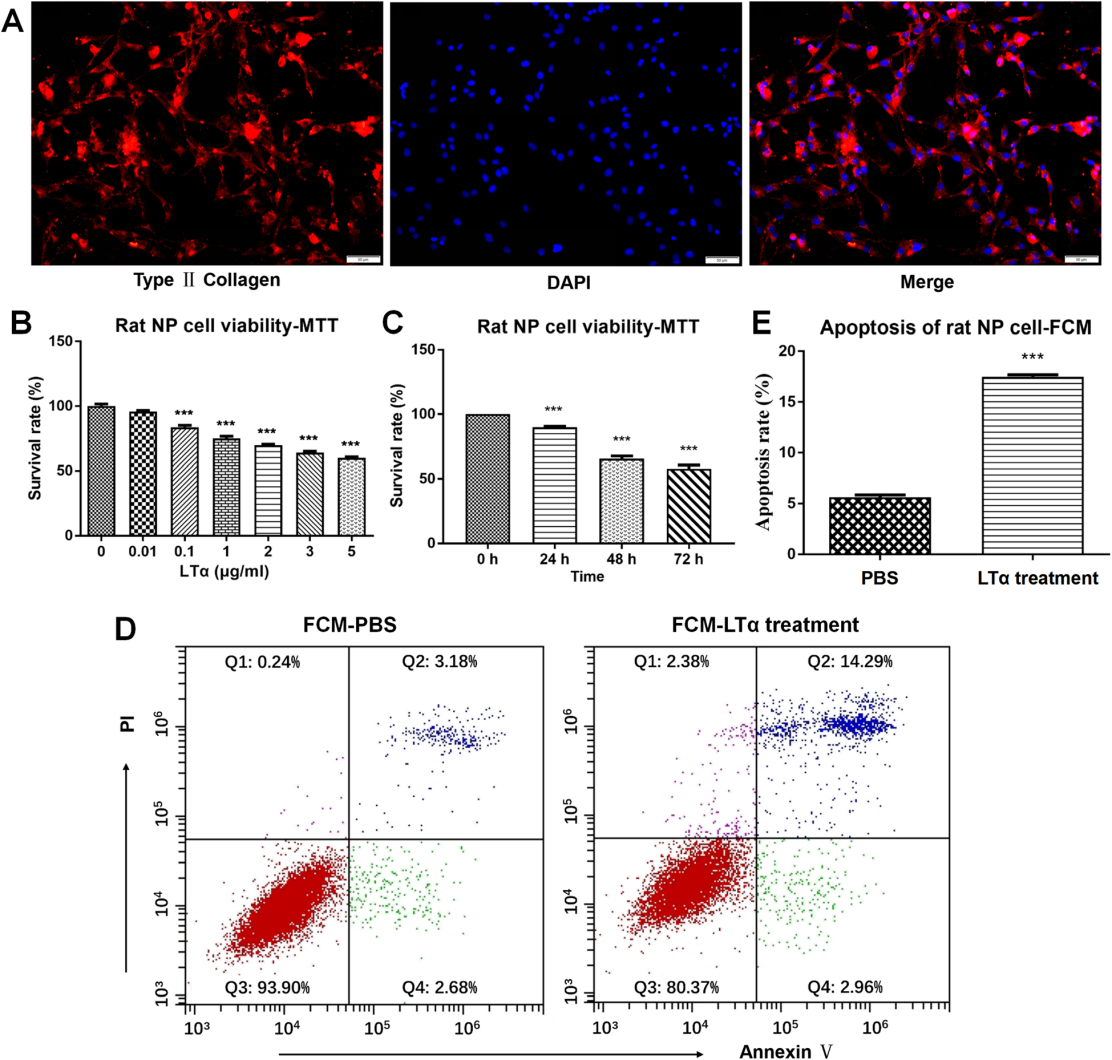
Immunofluorescence of primary NP cells and the effects of LTα on rat NP cell viability. (A) Immunofluorescence staining identified the rat primary NP cells. Red fluorescence: type II collagen-positive primary NP cells; blue fluorescence: DAPI-stained nuclei. The positive rate of NP cells was > 90%, and the purity of primary NP cells was > 90%. Scale bar: 50 μm. (B) Survival rate of rat NP cells that were treated or not with increasing concentrations of LTα for 72 h as determined by the MTT assay. (C) Survival rate of rat NP cells that treated or not with LTα (5 μg/ml) for various times as determined by the MTT assay. (D) LTα (5 μg/ml) and an equal volume of PBS were added to the experimental and control groups for 72 h, respectively, and the apoptosis rates of rat NP cells were determined by FCM. The apoptosis rate (Q2 + Q4) in the experimental group increased significantly (Q2: the late apoptotic stage; and Q4: the early apoptotic stage). (E) Statistical analysis of the apoptosis rate. ***, *p* < 0.001 (Unpaired, two-tailed Student’s *t*-test)

The purity of primary NP cells was > 90% as shown by type II collagen immunofluorescent staining (Fig. 3a). P3 NP cells in monolayer cultures were treated or not with increasing concentrations of LTα. After 72 h, cell viability was determined by MTT. The survival rate decreased in parallel with the increase in LTα concentration (Fig. 3b). NP cell viability was significantly decreased when LTα was 5 μg/ml. Subsequently, NP cells were treated or not with LTα (5 μg/ml) for various times. The survival rate decreased with time of cell exposure to LTα (Fig. 3c). A dose of LTα at 5 μg/ml and an exposure time of 48 ~ 72 h were defined as the optimal conditions for the LTα treatment to induce rat NP cell degeneration.

**Fig. 4:**
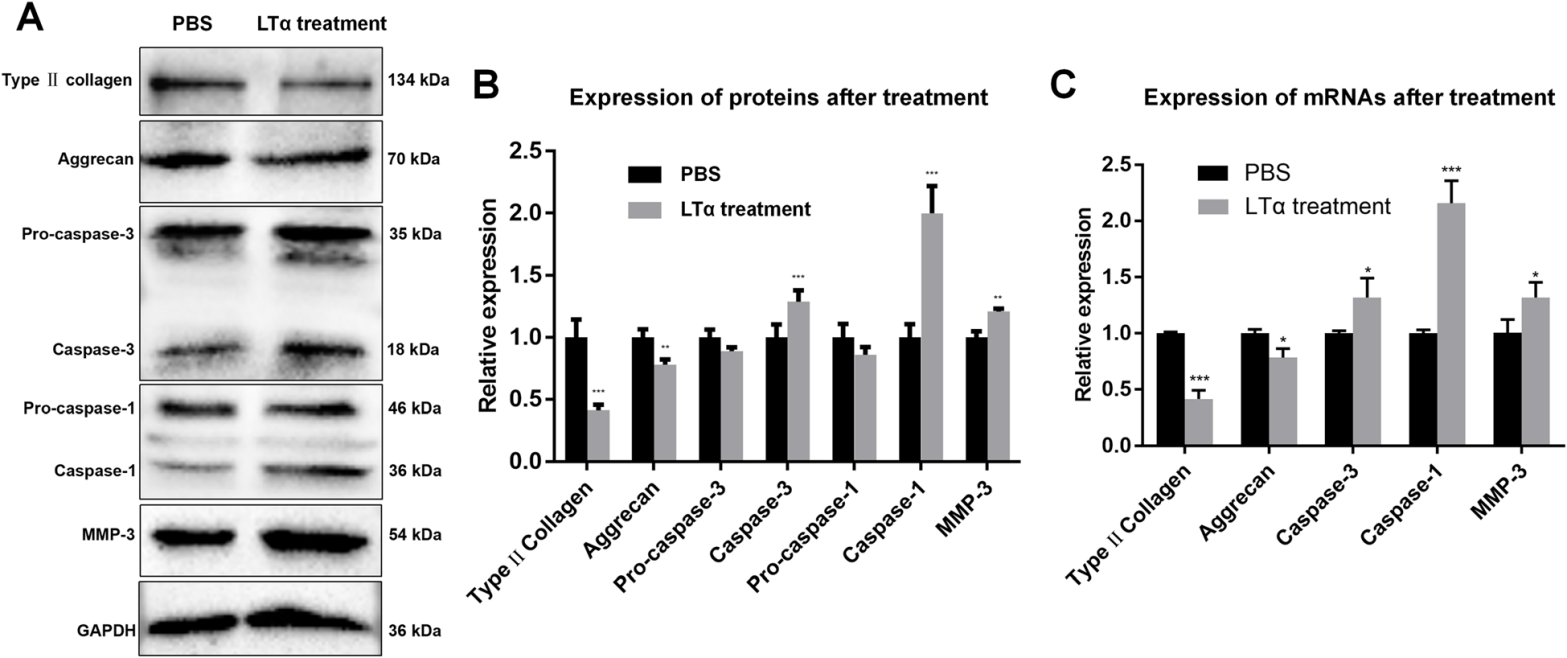
Effects of LTα on degeneration-related molecules in rat NP cells. (A) P3 NP cells were plated on 6-well culture plates. LTα (5 μg/ml) and an equal volume of PBS were added to the experimental and control groups for 72 h, respectively. Total proteins were extracted for the WB analysis. Full image WB images have been provided in Supplementary 1. (B) Quantitative analysis of protein levels as determined in “A”. (C) Treatment conditions were the same as that in “A”. Total mRNA was extracted for RT-qPCR analysis. *, *p* < 0.05; **. *p* < 0.01; ***. *p* < 0.001 (One-way ANOVA, Tukey’s multiple comparison analysis)

After the LTα treatment (5 μg/ml, 72 h), FCM, RT-qPCR and WB were used to evaluate the effect of LTα on NP cell apoptosis. The apoptosis rate (Q2 + Q4) was increased after the LTα treatment (Fig. 3d and e). The mRNA and protein expression levels of Caspase-3 and Caspase-1 also significantly increased (Fig. 4).

### LTα upregulated MMP-3 expression and downregulated the ECM protein levels

WB and RT-qPCR were used to determine the expression of degeneration-related molecules in NP cells in the control (PBS) and experimental (LTα treatment) groups. The mRNA and protein expression of MMP-3 was significantly increased in the experimental group, while that of type II collagen and aggrecan, the core components of ECM, were significantly decreased after the LTα treatment (Fig. 4).

## Discussion

The new findings of this study include the following: 1) LTα increased in the NP tissue associated with IVDD; and 2) LTα could induce NP cell apoptosis and reduce important ECM proteins, as indicated by in vitro testing. In addition, the optimal conditions for LTα treatment to induce rat NP cell degeneration was determined.

LTα is a member of the TNF superfamily and is one of the earliest discovered cytokines. Studies have demonstrated that LTα and TNFα have many similarities in gene structure, protein molecular structure, and biological functions; thus, LTα was formerly known as TNFβ [14]. However, further studies revealed many differences between TNFβ and TNFα, especially in cell origin, secretion dynamics, signal transduction pathway, and gene expression regulation. Both TNFα and LTα can bind to TNF-receptors (TNFR1 or TNFR2); however, when LTα exists in heterotrimer form (LTα_1_β_2_ or LTα_2_β_1_), it can also bind to another unique receptor, the LTβ receptor (LTβR) [24]. In addition, the herpesvirus entry mediator (HVEM) may also act as a receptor for LTα [13]. Among these receptors, activated TNFR1 can trigger an apoptotic cascade via TNFR-associated death domain (TRADD); TNFR2, LTβR, and HVEM link to intracellular signaling pathways via TNFR receptor-associated factors (TRAFs); and TNFR1 can also regulate TRAF2 via TRADD [13]. Because of stronger biological effects on tumor-killing activity and immunoinflammatory mediation, TNFα has received considerable amounts of attention [14]. In contrast, few studies have investigated the role of LTα, especially in IVDD pathology. In recent years, the significance of cytokine LTα in various diseases has been extensively investigated [15, 16]. These studies showed close relationships between LTα and immunoinflammatory-related diseases, such as RA and GVHD. Studies have also shown that LTα can activate the inflammatory environment in human chondrocytes [15] and NP cells commonly exhibit chondrocyte-like characteristics [25]. These potential connections inspired us to further study the relationship between LTα and IVDD. In this study and our previous work, the LTα level was significantly increased in degenerated human NP tissue, suggesting a potential correlation of LTα in the occurrence and development of IVDD [17].

Abnormal apoptosis of NP cells is an important cause of IVDD [26]. Excessive apoptosis of NP cells leads to decreased cell viability, which results in decreased synthesis of ECM. Caspase-3 is the most critical executive molecule in mediating apoptosis [27]. Caspase-1 is a crucial regulator of inflammatory mediator and has a vital role in the death receptor-mediated apoptotic pathway [28, 29]. We have shown that the apoptosis rates and the Caspase-3 and Caspase-1 levels were significantly increased after LTα treatment of NP cells. LTα can induce apoptosis of NP cells, which leads to the reduced synthesis of ECM, thereby suggesting a potential role of this process in the acceleration of IVDD development.

Most MMPs in discs are produced by NP cells and inner AF cells, and they are usually considered inactive zymogens in normal discs [30]. Cascade amplification effects occur when MMPs are activated, which leads to the degradation of the ECM. As a key enzyme in degrading disc ECM, MMP-3 not only directly degrades most proteoglycans, gelatins, and collagens but also activates other types of MMPs, thereby contributing to cascades and accelerating ECM degradation [9, 31]. In this study, MMP-3 was significantly upregulated in NP cells that were treated with LTα, and type II collagen and aggrecan, which are the core components of ECM, decreased. These findings indicate that the reduction in these key ECM proteins may lead to adverse effects on IVDD progress.

LTα reduced NP cell viability, and significant correlations were observed among the dose, treatment time, and cellular survival rate. These findings indicate that IVDD is a process wherein adverse effects associated with inflammatory cytokines accumulate. Within a certain range, a longer time of LTα exposure at higher LTα doses resulted in more severe NP cell degeneration. Thus, we concluded that the optimal conditions (dose and time) for LTα treatment to induce rat NP cell degeneration are 5 μg/ml and 48 ~ 72 h. TNFα-induced disc degeneration models have been widely utilized in previous studies [32, 33]; however studies presented limitations because of the severe toxicity of TNFα [34]. The toxicity of LTα is much lower than that of TNFα, indicating that LTα may have a potential value in the development of disc degeneration models [34].

Anti-TNFα therapy is effective in treating many immunoinflammatory diseases, including degenerative disc diseases and RA [35, 36]. Recent studies showed that up to 50% of RA patients were insensitive or even resistant to anti-TNFα treatment, while a combined treatment of anti-TNFα and anti-LTα could achieve RA remission [37, 38]. These cases not only indicated the value of anti-LTα therapy in immunoinflammatory diseases but also provided new insights and inspiration for a combined anti-TNFα and anti-LTα treatment for degenerative disc diseases. The similarities and differences between LTα and TNFα could complement and enrich each other in the treatment of diseases, thus achieving better therapeutic effects.

Several limitations were observed to this study. First, because fundamental research on the role of cytokine LTα in disease is limited at present, the theoretical basis of the relationship between LTα and degenerative diseases, especially LTα and IVDD, is still in the initial stage of exploration. The causality between the increase in LTα and the pathogenesis of IVDD remains unclear, and further studies are needed to explore the signal transduction pathways by which elevated LTα causes IVDD. In addition, the lack of a completely healthy disc source is the most critical restrictive factor in basic research on IVDD. Pathological and molecular changes have been found in scoliotic or spinal traumatic disc tissues [39, 40]. Nevertheless, when an absolutely healthy disc specimen is absent, a relatively healthy disc obtained from a patient with scoliosis or spinal trauma has been considered as an ideal normal control sample by researchers in many previous basic research studies [41, 42]. In addition, the issue of age differences between groups associated with limited clinical sample collections should not be ignored. An absolutely healthy specimen from a donor would make the results more accurate and reliable.

## Conclusions

Available evidence indicates that the increase in LTα is closely related to IVDD and LTα can induce NP cell apoptosis and reduce important ECM proteins, which may lead to adverse effects on IVDD progress. The results may provide insights on the pathogenic effects of the cytokine LTα on NP cells and IVDD. Moreover, the optimal conditions for LTα treatment to induce NP cell degeneration were found. These findings may facilitate a better understanding of the mechanisms of IVDD and help identify new therapeutic targets for degenerative disc diseases.

## Supplementary Information

**Additional file 1.**

## Data Availability

All data generated or analyzed during this study are available from the corresponding authors on reasonable request.

## Abbreviations

**IVDD**: intervertebral disc degeneration
**LTα**: lymphotoxin-α
**TNFβ**: tumor necrosis factor-β
**WB**: Western blotting
**ELISA**: enzyme-linked immunosorbent assay
**NP**: nucleus pulposus
**MTT**: methyl-thiazolyl-tetrazolium
**FCM**: flow cytometry
**ECM**: extracellular matrix
**TNF**: tumor necrosis factor
**RA**: rheumatoid arthritis
**MRI**: magnetic resonance imaging
**AF**: annulus fibrosus
**CRP**: C-reactive protein
**PBS**: phosphate buffer saline
**DAPI**: 4, 6-diamino-2-phenyl indole
**MMP**: matrix metalloproteinase
**RT-qPCR**: real-time quantitative polymerase chain reaction
**GVHD**: graft-versus-host disease
**TNFR**: TNF-receptor
**LTβR**: LTβ receptor
**HVEM**: herpesvirus entry mediator
**TRADD**: TNFR-associated death domain
**TRAF**: TNFR receptor-associated factor
**DMSO**: dimethyl sulfoxide
**OD**: optical density
