# Supplementary material for: Elevated lymphotoxin-α (TNFβ) is associated with intervertebral disc degeneration
Source: BMC Musculoskelet Disord. 2021 Jan 13;22:77. doi: 10.1186/s12891-020-03934-7 (PMC7807514; doi:10.1186/s12891-020-03934-7)
Supplement: Supplementary file 1 — Additional file 1. [file 12891_2020_3934_MOESM1_ESM.pdf]

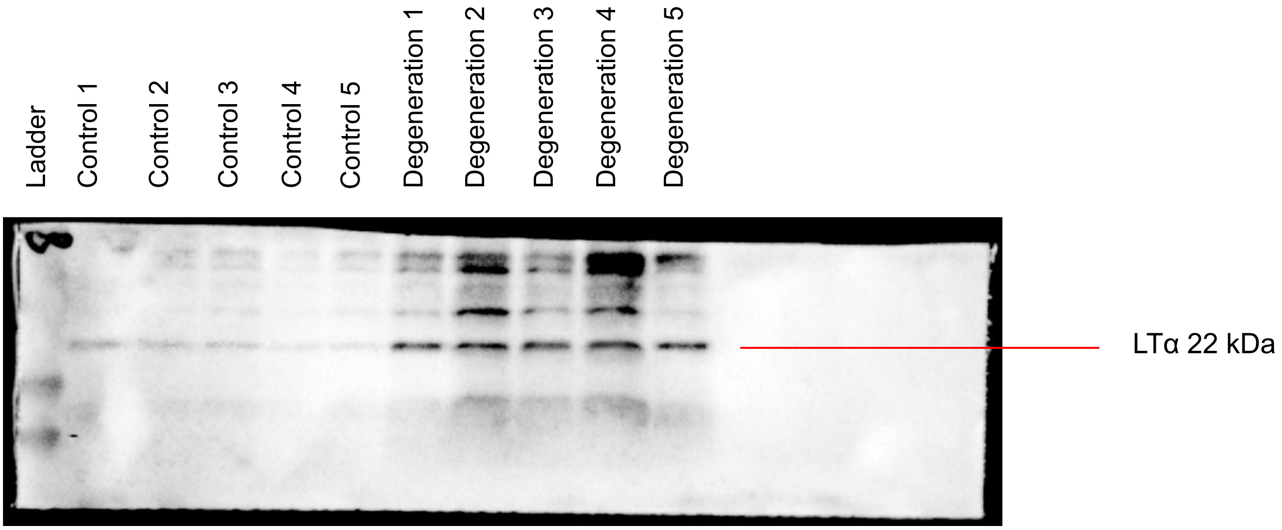

Exposure time: 10s

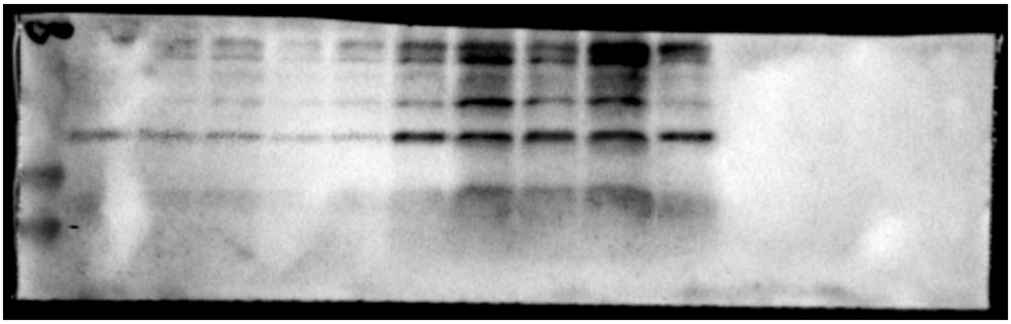

Exposure time: 20s

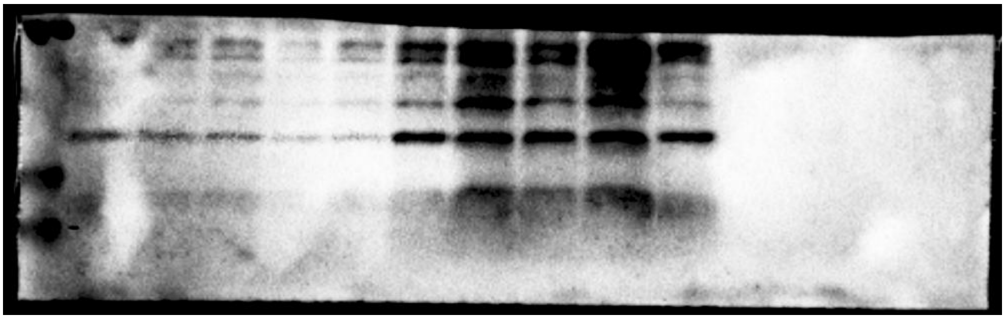

Exposure time: 30s

The bands of Control 5 and Degeneration 1 were selected as a typical image, as shown in Figure 1.

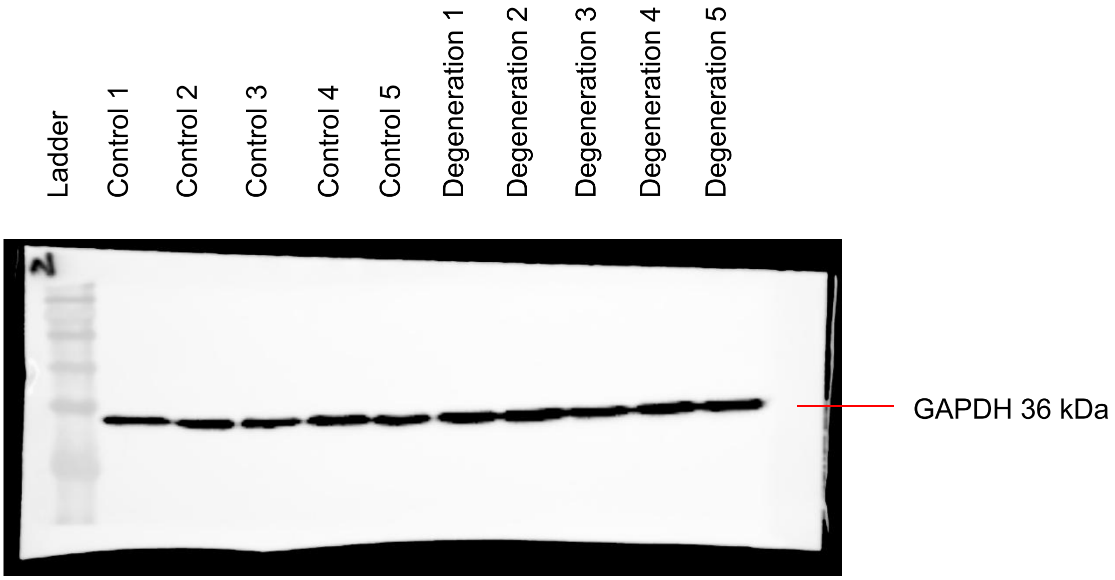

Exposure time: 10s

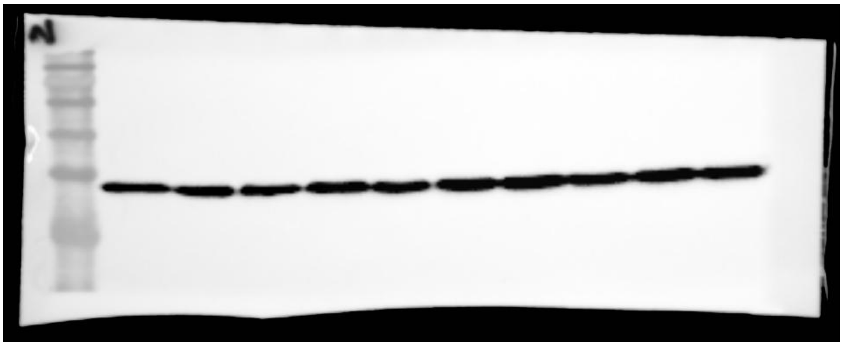

Exposure time: 20s

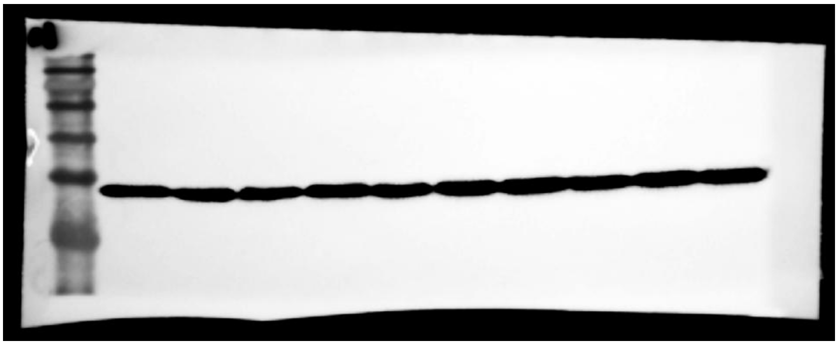

Exposure time: 30s

The bands of Control 5 and Degeneration 1 were selected as a typical image, as shown in Figure 1.

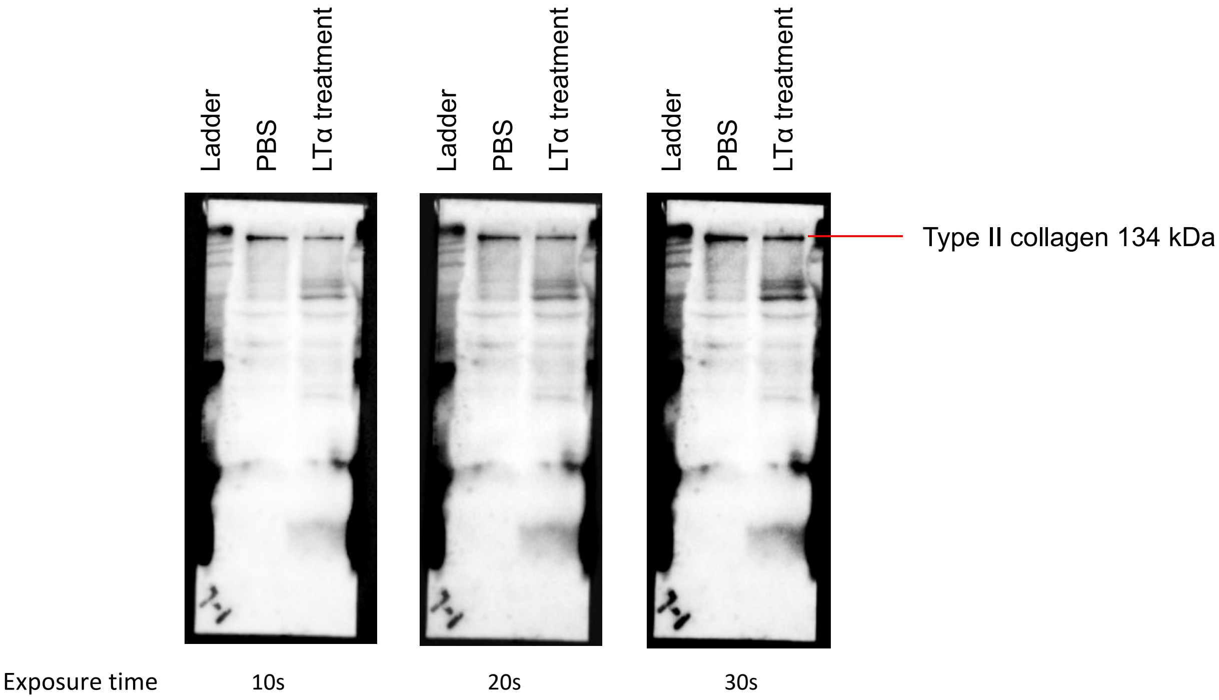
Ladder
PBS
LTα treatment
Ladder
PBS
LTα treatment
Ladder
PBS
LTα treatment
Type II collagen 134 kDa
Exposure time
10s
20s
30s

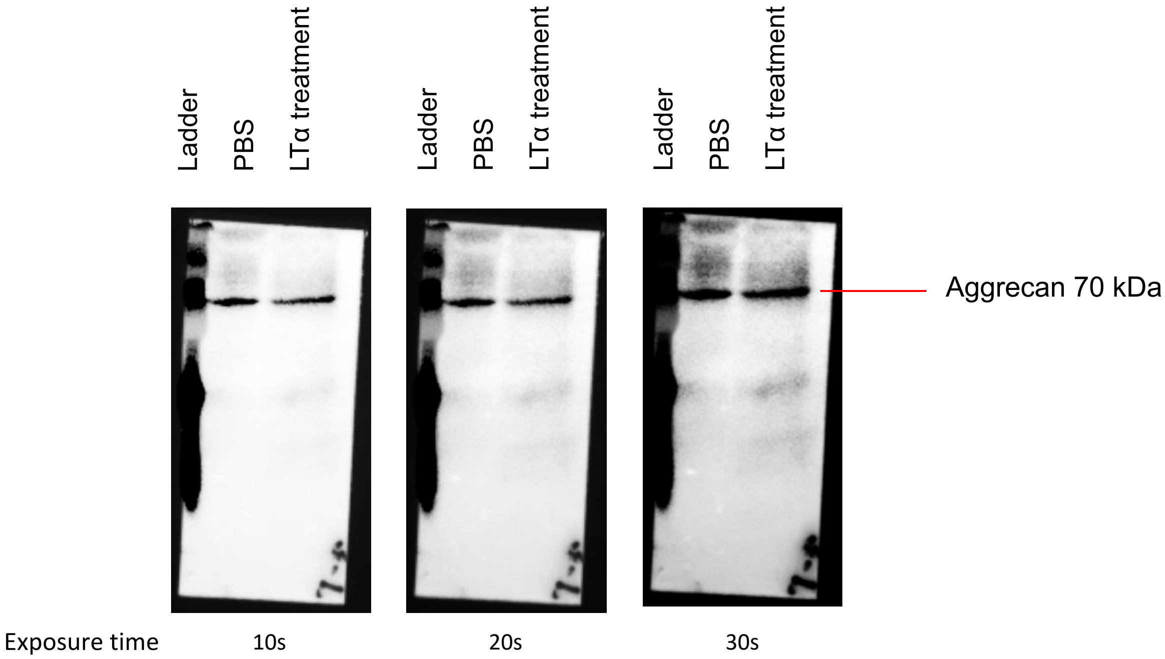
Ladder
PBS
LTα treatment
Ladder
PBS
LTα treatment
Ladder
PBS
LTα treatment
Aggrecan 70 kDa
Exposure time
10s
20s
30s

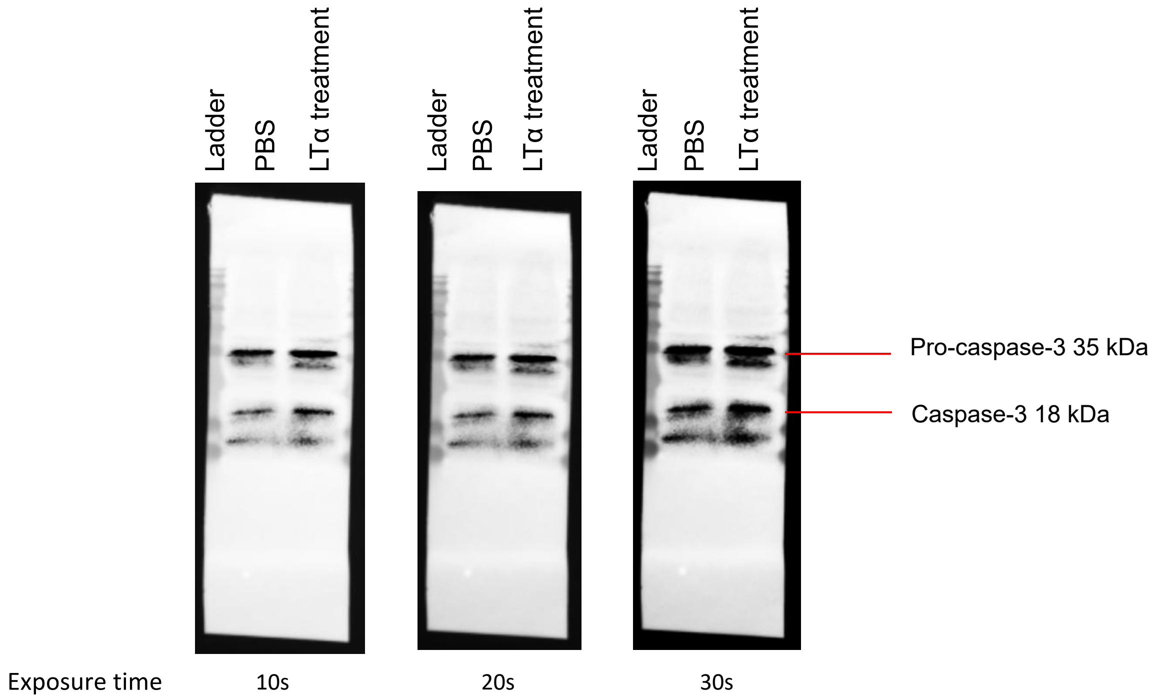
Ladder
PBS
LTα treatment
Ladder
PBS
LTα treatment
Ladder
PBS
LTα treatment
Pro-caspase-3 35 kDa
Caspase-3 18 kDa
Exposure time
10s
20s
30s

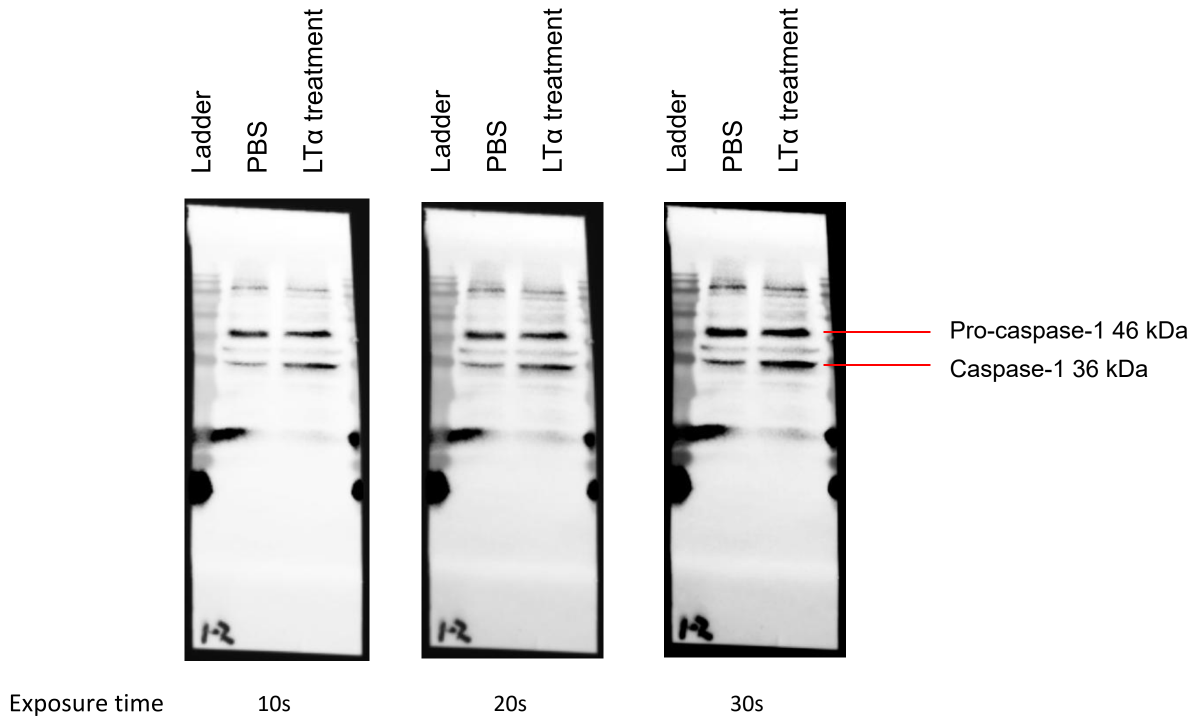
Ladder
PBS
LTα treatment
Ladder
PBS
LTα treatment
Ladder
PBS
LTα treatment
Pro-caspase-1 46 kDa
Caspase-1 36 kDa
1-2
1-2
1-2
Exposure time
10s
20s
30s

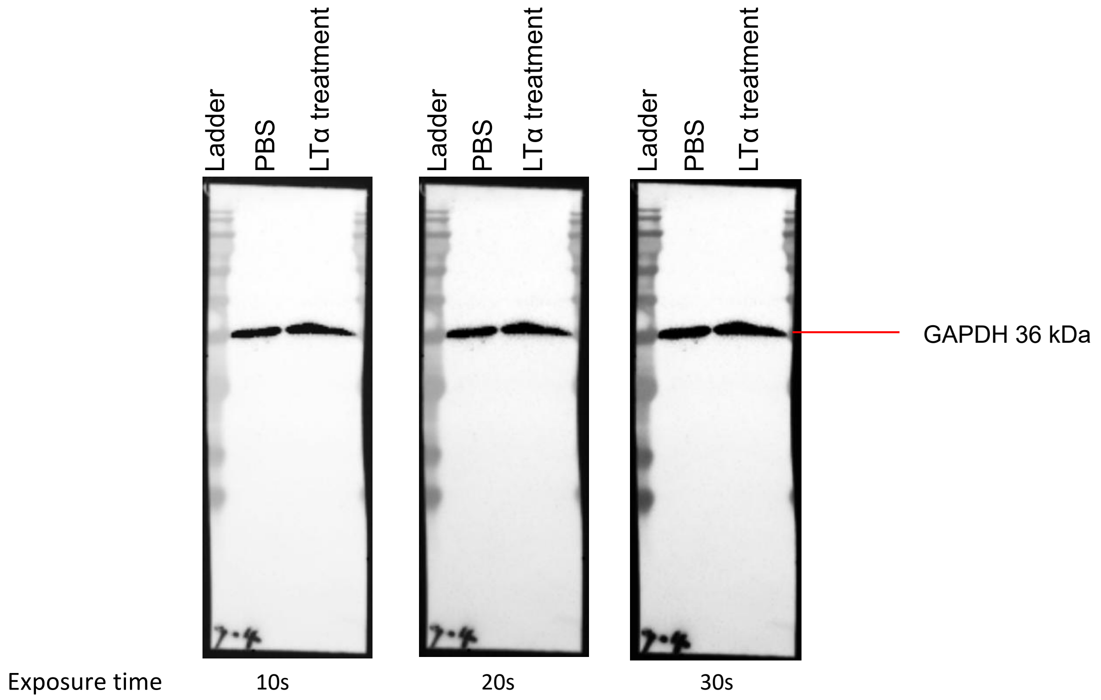
Ladder
PBS
LTα treatment
Ladder
PBS
LTα treatment
Ladder
PBS
LTα treatment
GAPDH 36 kDa
Exposure time
10s
20s
30s

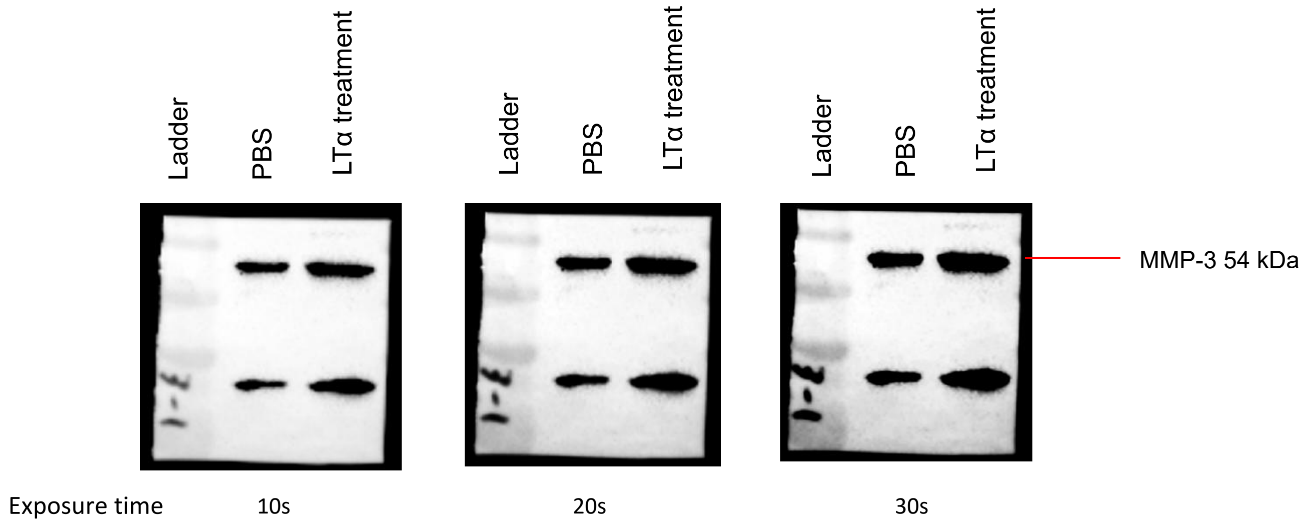
Ladder
PBS
LTα treatment
Ladder
PBS
LTα treatment
Ladder
PBS
LTα treatment
MMP-3 54 kDa
Exposure time
10s
20s
30s
